# Geographic heterogeneity of *Deinagkistrodon acutus* venom: A multi-regional study of toxicity, proteomics, and antivenom efficacy

**DOI:** 10.1371/journal.pntd.0014640

**Published:** 2026-08-11

**Authors:** Jianqi Zhao, Qian Lei, Xiaorui Hao, Xiao Shi, Ziqing Yang, Yuxin Luo, Linfeng Wang, Yang Yang, Chunhong Huang

**Affiliations:** 1 Department of Biochemistry and Molecular Biology, School of Basic Medical Sciences, Jiangxi Medical College, Nanchang University, Nanchang, Jiangxi, China; 2 Health Management Center, The Second Affiliated Hospital of Nanchang University, Nanchang, Jiangxi, China; 3 Queen Mary School, Jiangxi Medical College, Nanchang University, Nanchang, Jiangxi, China; Instituto Butantan, BRAZIL

## Abstract

Snakebite envenomation caused by *Deinagkistrodon acutus* is a severe public health problem. Growing evidence indicates that snake venom exhibits obvious intraspecific geographic variation, which directly affects toxicity, organ damage, and antivenom efficacy. However, systematic studies on the geographic heterogeneity of *D. acutus* venom remain insufficient. In this study, we collected venoms from different geographical populations of *D. acutus* in China and performed a comprehensive analysis of lethality, plasma biochemistry, coagulation function, histopathology, proteomics, and antivenom neutralization. The results showed that venoms from different regions exhibited significant differences in LD_50_. Histopathological examination revealed that the spleen and lung were the main target organs, and the severity of multi-organ injury varied greatly among regions. Proteomic analysis revealed that all venoms shared a core protein set, whereas the composition and abundance of phospholipase A_2_ and snake venom metalloproteinase exhibited marked geographic diversity, and these compositional differences were closely correlated with variations in venom potency. Importantly, commercial antivenom showed effective neutralization against venoms from eastern regions but significantly reduced protective efficacy against southern and western populations. In conclusion, *D. acutus* venoms from different geographical origins present remarkable heterogeneity in toxicity, organ pathogenicity, toxin composition, and antivenom reactivity. This study provides key evidence for understanding venom geographic variation and offers an important theoretical basis for optimizing clinical treatment and developing region-adapted antivenoms.

## Author summary

Snakebite envenomation by *D. acutus* is a severe public health problem in China, causing substantial morbidity and mortality. However, the venom of this species is known to exhibit significant geographic variation, which can profoundly impact clinical manifestations and the efficacy of standard antivenom treatments. Despite its clinical importance, a systematic, multi-regional investigation into this heterogeneity has been lacking. Here, we comprehensively analyzed the toxicity, proteomic composition, and antivenom neutralization profiles of *D. acutus* venoms collected from 14 distinct geographic regions across China. Our findings reveal that regional venom variations are closely correlated with differential abundance of key toxin families, particularly phospholipase A_2_ and snake venom metalloproteinases. Importantly, this geographic heterogeneity directly affects the neutralizing efficacy of the commercial antivenom. This study provides a molecular and pathological basis for understanding venom geographic variation, and offers critical evidence for the future development of region-adapted antivenoms to optimize clinical treatment of snakebite in China.

## Introduction

Snakebite envenomation represents a neglected tropical disease of global public health significance, causing an estimated 1.8–2.7 million envenomings and over 100,000 deaths annually worldwide, with the highest burden concentrated in rural regions of Asia, Africa, and Latin America [1,2]. In China, *Deinagkistrodon acutus* is one of the most medically important venomous snakes, widely distributed across regions in southern and central China. Bites from *D. acutus* frequently result in severe local tissue necrosis, systemic hemorrhage, multiple organ dysfunction syndrome, and even death, posing a substantial threat to the lives and health of residents in endemic areas [3,4].

A well-documented and clinically critical phenomenon in snake venom research is intraspecific geographic variation in venom composition and toxicity [5–9]. Snake venoms are complex mixtures of bioactive proteins and peptides, whose composition and function are shaped by long-term adaptive evolution to local ecological factors, including prey species, climate, habitat, and genetic differentiation [10,11]. For *D. acutus*, which spans a vast geographic range with diverse ecological environments, significant differences in venom toxicity, clinical manifestations, and antivenom responsiveness have been anecdotally reported in clinical practice [8,12]. Recent advances in proteogenomics‑guided functional venomics have provided the first holistic genome‑based overview of the toxin arsenal of *D. acutus*, predicting the molecular and functional basis of its life‑threatening effects [13]. However, to date, no systematic, multi-dimensional study has comprehensively characterized the geographic heterogeneity of *D. acutus* venom across its entire distribution range, leaving the molecular basis of venom variation and its clinical implications poorly understood.

Clinically, the primary treatment for snakebite envenomation is the administration of specific antivenom [14]. However, clinical retrospective studies and cross-regional antivenom comparison analyses have indicated that this monovalent antivenom exhibits variable neutralization efficacy against envenomation by snakes from different geographic regions [12,15]. Similar patterns of intraspecific geographic venom variation and its impact on antivenom efficacy have been documented in other viperid species. For instance, comparative proteomic analysis of Calloselasma rhodostoma venoms from Malaysia, Indonesia, Thailand, and Vietnam revealed significant regional differences in venom protein composition, particularly in SVMP, SVSP, and PLA_2_ abundance, which were associated with variable immunoreactivity and neutralization by the Thai monovalent antivenom [16]. Clinical evidence further supports that region-specific antivenoms may provide superior inhibitory activity against locality-matched venom profiles [17]. These findings underscore the broader relevance of understanding geographic venom heterogeneity for optimizing antivenom therapy across different regions. This discrepancy is hypothesized to stem from geographic variation in venom toxin composition, particularly the differential abundance of key toxic components such as phospholipase A_2_ (PLA_2_), snake venom metalloproteinases (SVMPs), and cytotoxins. Without a clear understanding of venom heterogeneity across regions, it is difficult to rationally guide the optimization of antivenom production strategies, particularly in the context of geographically tailored immunotherapy.

**Fig 1:**
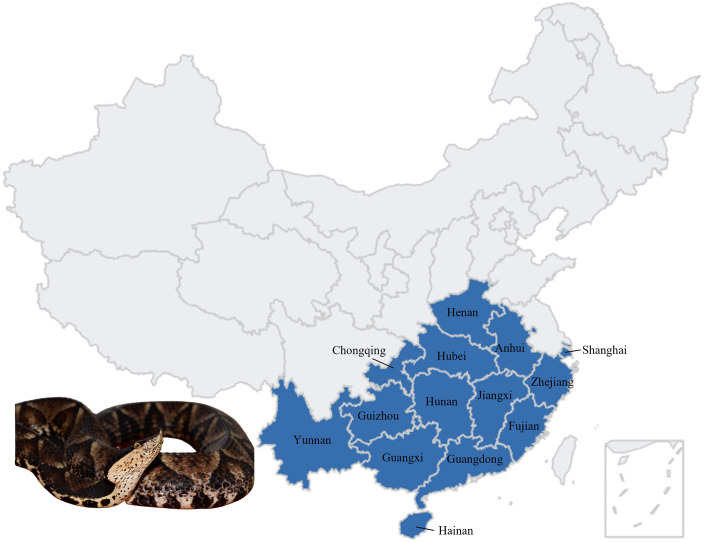
Geographical distribution of *D. acutus* sampling sites in this study. The blue-shaded areas on the map of China indicate the 14 provincial-level administrative regions where venom samples were collected, including Anhui, Chongqing, Fujian, Guangdong, Guangxi, Guizhou, Hainan, Henan, Hubei, Hunan, Jiangxi, Shanghai, Yunnan, and Zhejiang. The inset image shows the morphological characteristics of *Deinagkistrodon acutus*. Basemap: Natural Earth (https://www.naturalearthdata.com).

Against this background, we conducted a systematic, multi-dimensional investigation of *D. acutus* venoms from 14 distinct geographic regions across China (Fig 1). We integrated histopathological analysis, quantitative proteomics, toxin functional classification, and antivenom neutralization assays to address three core scientific questions: What are the differences in organ pathogenicity and toxicity profiles of *D. acutus* venoms from different regions? What is the molecular basis of geographic variation in venom composition, particularly the diversity of key toxin families? How does venom heterogeneity impact the efficacy of commercial antivenoms?

Our findings reveal significant geographic heterogeneity in the toxicity, organ damage, proteomic composition, and antivenom responsiveness of *D. acutus* venoms, with differential expression of PLA_2_ and SVMP as the core molecular driver of venom potency variation. This study not only advances our fundamental understanding of snake venom intraspecific evolution but also provides critical experimental evidence for optimizing clinical snakebite treatment, developing region-adapted or polyvalent antivenoms, and improving the prevention and control of snakebite envenomation in China.

## Materials and methods

### Snake venom collection and preparation

The *D. acutus* venom used in this study was sourced from the following regions in China: Anhui, Chongqing, Fujian, Guangdong, Guangxi, Guizhou, Hainan, Henan, Hubei, Hunan, Jiangxi, Shanghai, Yunnan, and Zhejiang. All venom collection procedures strictly adhered to ethical guidelines for the handling of wild animals and were performed by professionally trained personnel. Venom was extracted by allowing the snakes to bite through a cellulose-covered membrane into sterile 50 mL centrifuge tubes. For each geographic region, venom samples from individual snakes of the same species were pooled. After lyophilization, the pooled venom samples were stored at –20 °C until further use [18]. Venom was collected from 5 adult individuals per region, all sampling was uniformly performed in July (when snakes are physiologically most active), and all snakes were confirmed as adults by three nationally certified professional in the breeding of *D. acutus*.

### Animal models and ethics

Kunming mice (male, weighing approximately 25–35 g, 6–8 weeks old) were obtained from the Animal Center of Nanchang University (Nanchang, Jiangxi Province, China). According to previously described experimental procedures [19], each group consisted of six mice, and a total of fifteen groups were included in this study: (a) normal saline (control group); (b) Anhui; (c) Chongqing; (d) Fujian; (e) Guangdong; (f) Guangxi; (g) Guizhou; (h) Hainan; (i) Henan; (j) Hubei; (k) Hunan; (l) Jiangxi; (m) Shanghai; (n) Yunnan; and (o) Zhejiang. The administered dose was set at 0.2 times the median lethal dose (LD_50_) of the venom. At 24 hours post-injection via intraperitoneal administration routes, blood samples were collected from the periorbital region using retro-orbital puncture under isoflurane anesthesia. The blood was transferred to heparin-coated tubes and centrifuged at 3,000 rpm for 10 minutes at 4 °C. Plasma aliquots were stored at –80 °C. Subsequently, the mice were euthanized by cervical dislocation, and major organs were harvested. Mouse plasma and organ tissues were utilized for subsequent experiments. All animal experiments were conducted in strict accordance with the guidelines for animal experimentation at Nanchang University and were approved by the Animal Ethics Committee of Nanchang University (Ethical Code: 20220624042).

### LD_50_ determination

The median lethal dose was evaluated via intraperitoneal injection in male mice, with six animals allocated per dose group. Venom samples from each species were prepared in sterile normal saline (0.9% NaCl) and subjected to serial dilution, with concentrations ranging from 1 to 30 mg/kg body weight for *D. acutus*. Mice received a constant injection volume of 0.1 mL per dose. Mice in the control group received an equivalent volume of normal saline solution. Mortality was assessed 24 hours post-administration. LD₅₀ values were calculated using the Spearman–Karber method [18].

### Antivenom neutralization assay

Commercial monovalent *D. acutus* antivenom was used in this study. The neutralization assay was performed using an in vitro pre-incubation method. To ensure standardized inter-regional comparison, a uniform dose of antivenom with identical total protein content was used across all regional groups, which was set at 2-fold the protein amount of Anhui venom at the 4 × LD_50_ challenge dose. Briefly, for each geographic venom group, 4 × LD_50_ of regional venom was thoroughly mixed with the fixed-dose antivenom, and the mixture was pre-incubated at 37°C for 30 min. Subsequently, the venom-antivenom mixture was administered via intraperitoneal injection to mice (n = 6 per group). Mice in the control group received an equal volume of venom mixed with normal saline instead of antivenom. Survival status of all animals was monitored continuously for 8 h, and survival curves were generated accordingly. All experimental procedures were fully standardized across all regional groups to ensure comparability of results.

### Blood cell count analysis

Blood samples collected from the experimental mice were directly introduced into a fully automated hematology analyzer for quantitative evaluation of hematological parameters. Leukocyte counts and other cellular indices were determined in strict accordance with the manufacturer's standardized operating procedures to ensure accuracy, reliability, and reproducibility of results throughout the study.

### Plasma enzyme assay

Plasma samples collected in the preceding experiments were subjected to enzymatic analysis using commercial assay kits. The biochemical parameters measured included alanine aminotransferase (ALT), aspartate aminotransferase (AST), creatine kinase (CK), and plasma creatinine (Scr). All procedures were performed in strict accordance with the manufacturer's provided protocols.

### Coagulation parameter assessment

Hemostatic parameters, including fibrinogen concentration (FIB), prothrombin time (PT), thrombin time (TT), and activated partial thromboplastin time (APTT), were measured using commercially available kits. All analyses were performed on a Servicebio fully automated coagulation analyzer in strict accordance with the manufacturer's instructions.

### Histology

Histological examination was performed according to established protocols with minor modifications [11]. Harvested tissue specimens were fixed by immersion in 4% paraformaldehyde for at least 24 hours, dehydrated through a graded ethanol series, and embedded in paraffin. Sections were cut at a thickness of 5 μm. Following deparaffinization in xylene, the sections were stained with hematoxylin and eosin (H&E) for microscopic evaluation.

### SDS-PAGE

For SDS-PAGE analysis, equal amounts of total protein (10 μg per lane) were loaded for all venom samples. Protein concentrations were determined using the BCA assay prior to electrophoresis, and the loading volume for each sample was calculated accordingly. Electrophoresis was performed on 12% sodium dodecyl sulfate-polyacrylamide gels under reducing conditions. Following electrophoresis, gels were stained with Coomassie Brilliant Blue G-250 for 1 hour and destained overnight in a methanol-acetic acid solution. All gels were stained under identical conditions and imaged using the same instrument with uniform illumination settings to ensure consistency across samples and to minimize staining‑related variability.

### LC-MS/MS preparation

Lyophilized venom protein (100 µg) was dissolved in 10 mM dithiothreitol (DTT) and incubated at 55 °C for 30 min to reduce disulfide bonds. After cooling the mixture to room temperature on ice, alkylation was performed by adding 55 mM iodoacetamide (IAA) and incubating in the dark for 15 min. Proteins were precipitated by adding six volumes of pre-chilled acetone and stored at –20 °C for at least 4 h. The precipitate was collected by centrifugation (8,000 rpm, 10 min, 4 °C) and briefly air-dried (2–3 min) to remove residual acetone. The pellet was dissolved in 100 µL of 50 mM ammonium bicarbonate (NH_4_HCO_3_) and digested overnight at 37 °C with sequencing-grade trypsin (1 mg/mL, enzyme-to-protein ratio of 1:100, w/w). Peptides were desalted using 96-well plates (Thermo Fisher, Waltham, MA, USA). The columns were activated three times with 200 µL of methanol and equilibrated with 200 µL of 0.1% formic acid in water. The sample solution (500 µL) was loaded under vacuum at approximately 1 mL/min, washed three times with 200 µL of 0.1% formic acid, and eluted three times with 150 µL of 50% acetonitrile/0.1% formic acid. The combined eluates (450 µL) were dried in a vacuum concentrator prior to LC-MS/MS analysis [18,20,21].

### LC-MS/MS analysis

Prior to analysis, indexed retention time (iRT) standards were spiked into the triplicate digests of each species at a ratio of 1:20 (v/v). Equal amounts of peptides were separated on a C18 analytical column (Thermo Fisher Scientific) using mobile phase A consisting of water with 0.1% formic acid and mobile phase B consisting of acetonitrile with 0.1% formic acid at a flow rate of 400 nL/min. The gradient program was set as follows: 5–22% B (0–20 min), 22–37% B (20–24 min), 37–80% B (24–27 min), and 80% B (27–30 min). Mass spectrometric analysis was performed on a timsTOF Pro system (Bruker) with the following parameters: capillary voltage, 1.4 kV; dry gas flow rate, 3.0 L/min (180 °C); ion mobility range, 0.7–1.3 V·s·cm^2^; mass scan range, m/z 100–1700; and collision energy range, 20–59 eV. Data-independent acquisition (DIA) data were searched against the UniProt_Viperidae venom database using Spectronaut Pulsar (v18.4) software. The MS raw data were processed using MaxQuant (version 2.0.3.0). For database searching, the precursor ion mass tolerance was set to 20 ppm, and the fragment ion mass tolerance was set to 0.05 Da. The search was performed against the NCBI Serpentes database (or the relevant proteome database used). A maximum of two missed tryptic cleavages was allowed. Protein identification required a minimum of one unique peptide, and results were validated using the Morpheus scoring system. Redundant peptides were removed by homology-based sequence alignment, and quantification was performed at the MS/MS level using extracted fragment ion chromatograms [21–23]. To minimize the influence of missing values and stochastic protein detection on regional comparisons, proteins identified by fewer than two unique peptides or detected in fewer than 50% of samples within any geographic group were excluded. Only proteins consistently detected across biological triplicates were retained for downstream analysis.

### Statistical analysis

Data were expressed as mean ± standard deviation (SD). Statistical comparisons were conducted using one-way ANOVA followed by Tukey's post hoc test when appropriate (SPSS version 23.0, IBM Corporation, USA). A p-value < 0.05 was considered statistically significant.

## Results

### Geographic variability in venom-induced toxicity and antivenom neutralization

**Fig 2:**
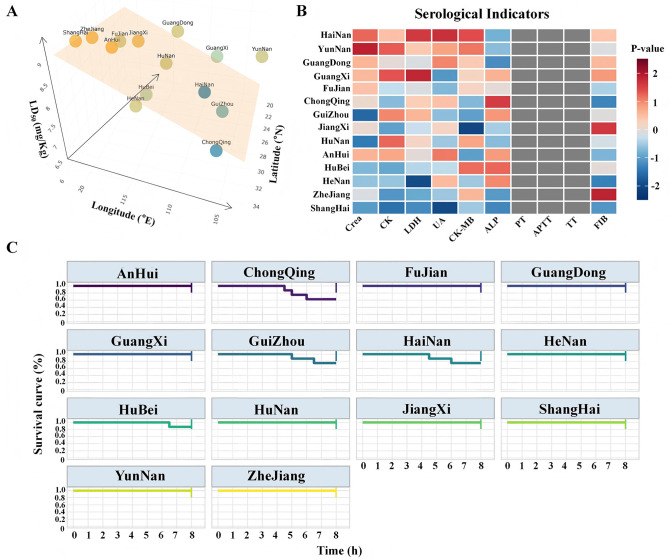
In *vivo* toxicological characterization of venoms from different geographic regions. **(A)** Relationship between the geographic coordinates (latitude and longitude) of *D.acutus* venom collection sites and the LD_50_ of the venom. **(B)** Clustered heatmap illustrating changes in plasma biochemical and coagulation parameters in mice following injection with venoms from different regions, including Crea, CK, LDH, UA, CK-MB, ALP, PT, APTT, TT, and FIB. **(C)** Survival curves of envenomed mice treated with antivenom.

To evaluate the relationship between geographic origin and venom-induced pathophysiology, we systematically assessed the lethal potency, systemic biochemical disruption, and antivenom efficacy across 14 distinct *D. acutus* populations. The result revealed pronounced geographic variability in *D.acutus* venom potency and pathophysiology. Venom lethality exhibited a distinct spatial trend across the 14 sampled regions, with higher toxicity generally observed in venoms from lower-latitude populations such as Hainan and Yunnan, whereas venoms from central and eastern provinces displayed comparatively reduced lethal potency (Fig 2A). Hierarchical clustering of plasma biochemical and coagulation parameters further stratified the venoms into distinct functional groups (Fig 2B). Notably, venoms from southern regions induced more severe myotoxic and cytotoxic profiles, reflected by elevated CK, CK-MB, and LDH levels, while all the venom causes similar and significant impairment of consumptive coagulation function.

These functional divergences directly impacted therapeutic outcomes. Survival analysis demonstrated that the commercial antivenom afforded complete protection against envenomation by venoms from several eastern populations; however, its efficacy was markedly diminished against venoms from southernmost and western regions (e.g., HaiNan, ChongQing, GuiZhou and HuBei), where survival rates dropped significantly despite treatment (Fig 2C). Collectively, these findings underscore that regional venom variation profoundly influences both clinical manifestation and antivenom neutralization capacity.

### Organ pathological injury profiles of snake venoms from different regions

**Fig 3:**
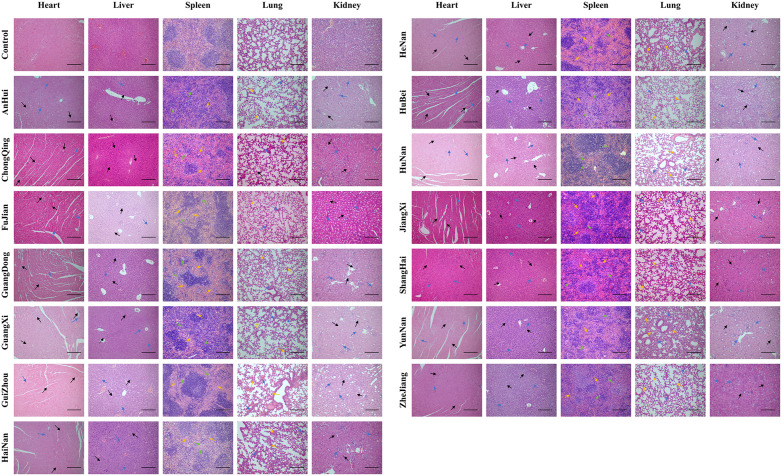
Histopathological changes of major organs in mice exposed to snake venoms from different geographical origins. Heart, liver, spleen, lung, and kidney tissues were stained with hematoxylin and eosin. In the histopathological images, black arrows indicate swollen and necrotic cells, blue arrows denote inflammatory cell infiltration, yellow arrows point to erythrocyte infiltration, and green arrows highlight the starry sky pattern formed by macrophage phagocytosis. Compared with the control group, mice exposed to various snake venoms exhibited distinct histological injuries, with the spleen and lung being the most severely affected target organs. Venoms from ChongQing, FuJian, JiangXi, and ShangHai caused more severe multi-organ damage, while venoms from AnHui, GuangXi, GuiZhou, YunNan, and ZheJiang induced relatively mild pathological alterations. Scale bar: 200 μm.

After snake venom enters the body, it typically causes severe organ damage. To evaluate whether snake venoms from different geographical origins exhibit differences in organ toxicity, we performed a histopathological assessment. The results showed that (Fig 3), compared with the control group, all venom-exposed groups presented varying degrees of histological organ damage, with significant venom heterogeneity in both the injury spectrum and severity. Among all organs, the spleen and lung were the most consistently and severely affected target organs across all groups: the spleens of venom-exposed mice generally exhibited disorganized and blurred boundaries between the white pulp and red pulp, accompanied by massive lymphocyte proliferation and focal aggregation. Focal splenic necrosis and inflammatory cell infiltration were observed in mice treated with venoms from certain regions, such as Anhui, Guangxi, Jiangxi, and Shanghai. The lungs displayed typical pathological features of interstitial pneumonia, characterized by marked thickening of the alveolar septa, inflammatory cell infiltration, and alveolar collapse and fusion. Moreover, mice in the Hainan, Guizhou, Jiangxi, and Shanghai groups showed severe injury with massive inflammatory exudation in the alveolar spaces and pulmonary consolidation.

In contrast, the damage to the heart, liver, and kidney was distinctly venom-dependent. Venoms from Chongqing, Fujian, Jiangxi, and Shanghai induced severe multi-organ injury, including disorganized and fractured myocardial fibers with interstitial edema, marked eosinophilic change and vacuolar degeneration of hepatocytes, and in some cases (e.g., Fujian venom), renal tubular epithelial cell necrosis and renal interstitial inflammatory infiltration. Conversely, venoms from Anhui, Guangxi, Guizhou, Yunnan, and Zhejiang caused relatively mild injury to the liver and kidney, manifesting as mild interstitial edema, sparse inflammatory cell infiltration, and no large-scale tissue necrosis. In conclusion, snake venoms from different geographical origins show significant heterogeneity in organ pathogenicity, with the spleen and lung as the primary target organs post-exposure. These findings provide critical pathological evidence for subsequent studies on venom potency classification and pathogenic mechanisms of snake venom.

### Proteomic characteristics of snake venoms from different regions

**Fig 4:**
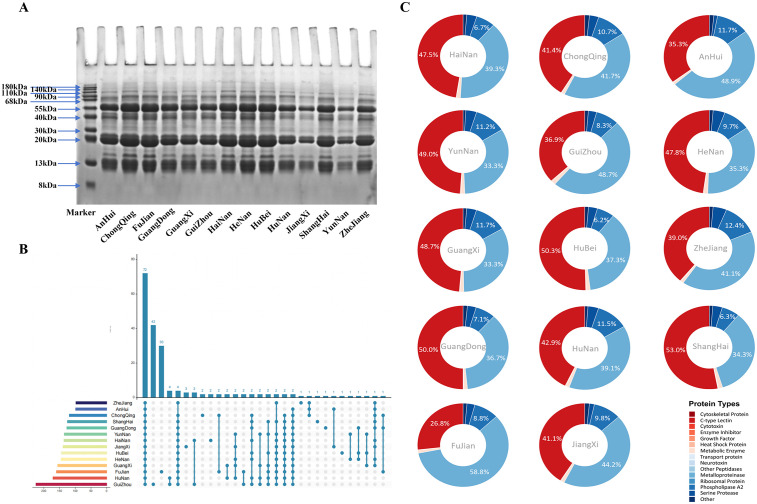
Proteomic characterization of snake venoms from different geographical origins. **(A)** SDS-PAGE analysis of venom proteins from 14 regions, with molecular weight markers indicated on the left. **(B)** The intersection of identified proteins across different regional venom groups, illustrating the shared and unique protein components among geographical isolates. **(C)** The protein composition profiles of snake venoms from each region, categorized by functional protein types.

Snake venom potency is closely associated with its component composition. SDS‑PAGE analysis showed that the overall protein band patterns of snake venoms from different geographical origins were relatively conserved, whereas significant differences were observed in band intensity (Fig 4A). UpSet plot analysis revealed the presence of a core set of shared proteins among all geographical venom isolates, as well as a large number of region‑specific unique proteins, indicating remarkable proteomic diversity across different regions (Fig 4B). Furthermore, protein composition analysis demonstrated that although the major functional toxin categories were similar among all venoms (Fig 4C), the relative proportions of key toxic components, including metalloproteinases, phospholipase A_2_, and cytotoxins, varied substantially between regions. Venoms from certain regions contained higher abundance of potent toxic proteins, which was consistent with the more severe organ damage observed in histopathological analysis. Collectively, these findings indicate significant proteomic heterogeneity among geographically distinct snake venoms, which may serve as an important molecular basis for the differences in their organ-targeting venom potency.

**Fig 5:**
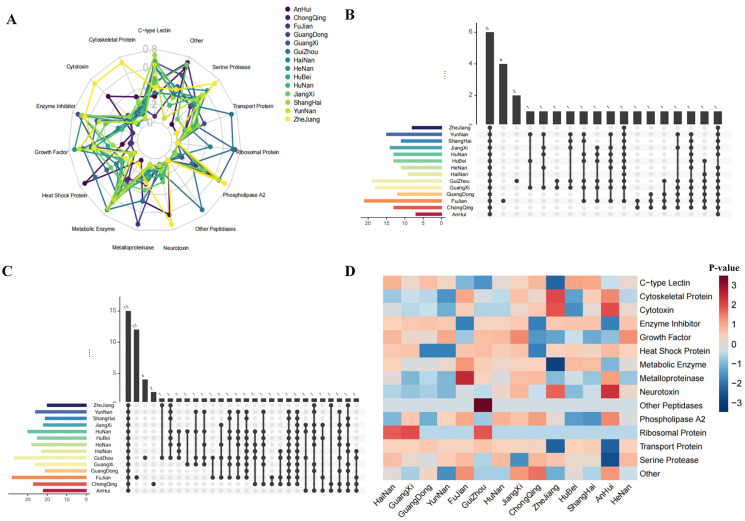
Main component analysis of snake venoms from different geographical origins. **(A)** The relative abundance of protein categories across different regional venom, highlighting the compositional differences in toxin profiles. **(B-C)** The intersection of PLA_2_ or SVMP isoforms in venoms from different regions. **(D)** Heatmap displaying the normalized abundance of key functional toxin categories in each regional venom, with red indicating high expression and blue indicating low expression.

To further explore the molecular basis of venom potency heterogeneity, we performed a systematic analysis of key functional toxin components. As shown in Fig 5A, the radar chart revealed distinct toxin composition profiles across different regional venoms, with significant differences in the relative abundance of core toxic components such as phospholipase A_2_, metalloproteinase, and cytotoxin. UpSet plots for phospholipase A_2_ (Fig 5B) and metalloproteinase (Fig 5C) further demonstrated that while a core set of shared isoforms existed across all regions, a large number of region-specific unique isoforms were identified, indicating remarkable diversity in these key toxin families. The heatmap (Fig 5D) confirmed these compositional differences, showing that venoms from high-venom potency regions exhibited significantly higher relative abundance of phospholipase A_2_ and metalloproteinase, which was consistent with their severe organ pathogenicity observed in histopathological assays. Collectively, these results suggest that the heterogeneity in the composition and abundance of key toxin families, particularly PLA_2_ and SVMP, may contribute to the geographical variation in venom potency, although direct isoform‑specific functional validation is warranted in future studies.

## Discussion

The present study systematically investigated the geographic variation in venom composition, organ pathological damage, in vivo toxicity, and antivenom efficacy of *D. acutus* from 14 regions in China, combined with proteomic and histopathological approaches. Our results demonstrated significant geographic heterogeneity in venom potency, organ pathogenicity, key toxin components, and therapeutic responses, providing critical insights into the intraspecific variation of *D. acutus* venom and its clinical implications.

Geographic variation in snake venom composition and toxicity is a common evolutionary phenomenon shaped by genetic differentiation, prey diversity, climate, and habitat differences [5,9,20,24]. However, the relationship between climate and venom potency is not unidirectional; while some studies have reported increased venom toxicity or enzymatic activity in warmer climates [25], others have documented higher venom potency in cooler or drier regions [26]. In this study, LD50 values varied markedly among *D. acutus* populations, consistent with the notion that regional ecological conditions—rather than temperature alone—shape venom phenotypes. Plasma biochemistry and coagulation assays further revealed that all venoms caused significant hemostatic disturbance, while southern populations induced more severe myotoxic and cytotoxic damage, reflected by elevated levels of CK, CK-MB, and LDH. These functional differences may reflect adaptive evolution of venom profiles to local prey species and environmental pressures, leading to diversified toxic phenotypes across geographic regions.

Histopathological analysis confirmed that spleen and lung were the primary target organs injured by *D. acutus* venom, manifesting as interstitial pneumonia, structural destruction of splenic white pulp and red pulp, and massive inflammatory infiltration [11,19]. Heart, liver, and kidney damage showed strong geographic dependence: venoms from Chongqing, Fujian, Jiangxi, and Shanghai caused severe multi-organ lesions including myocardial fiber disruption, hepatocellular degeneration, and renal tubular necrosis. This organ-specific pathogenic heterogeneity provides a pathological basis for understanding clinical differences in symptoms following bites by geographically distinct *D. acutus*. It is important to emphasize that while the histopathological data unequivocally demonstrate necrotic tissue damage, the elevated plasma activities of CK, CK‑MB, and LDH should be interpreted as indicators of systemic stress or increased membrane permeability, rather than as definitive markers of necrosis per se. Consequently, the integration of histological and biochemical evaluations provides a more holistic view of venom‑induced organ injury.

Proteomic analysis further revealed the molecular foundation underlying venom phenotypic variation [27,28]. SDS-PAGE showed overall conserved protein patterns among populations, while band intensity differed significantly, indicating quantitative variation in major toxin components. UpSet analysis demonstrated the existence of a core set of conserved proteins shared by all venoms, as well as abundant region‑specific unique proteins, reflecting both evolutionary conservation and geographic diversification (of course, we acknowledge that inherent limitations of proteomic workflows may have influenced some of our findings). Notably, PLA_2_ and snake venom metalloproteinase (SVMP) exhibited high isoform diversity and distinct abundance patterns across regions. Venoms with stronger toxicity and more severe organ damage contained higher proportions of PLA_2_ and SVMP, two major toxin families responsible for hemorrhage, tissue necrosis, inflammation, and coagulopathy [29,30]. Consistent with the well-documented pathogenic roles of snake venom PLA2 and SVMP in inducing hemorrhage, tissue necrosis, inflammatory injury and coagulation dysfunction, the regional differences in their abundance were highly concordant with the variation in LD₅₀, plasma biochemical markers and histopathological injury severity across populations, supporting that differential expression of these two toxin families is the core molecular basis of geographic venom potency variation. These findings indicate that compositional and quantitative differences in key toxins are the primary molecular drivers of geographic variation in *D. acutus* venom pathogenicity. It should be noted that our proteomic data reveal compositional and abundance differences at the protein family level, but do not directly establish isoform‑specific pathogenic activities. Future studies employing purified toxin isoforms and functional enzymatic or in vivo assays are needed to definitively link individual PLA_2_ and SVMP isoforms to specific pathological outcomes.

A clinically important finding is that geographic variation directly impairs the neutralization efficacy of commercial antivenom [31,32]. The antivenom provided complete protection against venoms from eastern populations but showed significantly reduced efficacy against those from southern and western regions (defined as 100% survival post-injection), including Hainan, Chongqing, Guizhou, and Hubei., although it did not necessarily prevent all non-lethal local or hemorrhagic effects. This observation highlights a critical limitation of current monovalent antivenoms, which are usually produced using venoms from limited geographic origins. Given the pronounced intraspecific venom divergence, antivenoms based on single-region venom may fail to provide optimal protection against bites by snakes from other regions. Our data support the necessity of developing geographic-adapted or polyvalent antivenoms that cover the toxin diversity of *D. acutus* across its distribution range.

There are several limitations in this study. First, the sex ratio and individual body size of sampled snakes were not systematically recorded, which remain potential uncontrolled variables when interpreting geographic venom variation. Second, the specific pathogenic mechanisms of key differential toxins and their synergistic effects require further verification through in vitro functional assays and molecular interaction studies [33–35]. Third, the in vivo mouse model may not fully recapitulate the envenomation process in humans, and clinical observations are needed for validation. Fourth, this study did not perform in vitro enzymatic activity assays for PLA2 and SVMP or targeted functional validation of individual toxin isoforms. The causal relationship between toxin abundance and pathological phenotypes requires further verification by dedicated functional experiments in future research. Furthermore, formal correlation analysis between toxin family abundance and specific pathological parameters was not performed in this study. The association between PLA2/SVMP differential expression and venom toxicity variation is inferred from consistent inter-regional phenotypic trends, which requires further statistical verification in dedicated mechanistic research. Additionally, our evaluation of antivenom neutralization efficacy was primarily based on survival analysis. While this approach is standard in preclinical antivenom assessment, it does not fully capture the extent of protection against local tissue damage, hemorrhage, or histopathological alterations. Comprehensive evaluation of antivenom efficacy would benefit from additional functional, histopathological, and molecular assessments in future studies.

In conclusion, *D. acutu*s venoms from different geographic regions exhibit strong heterogeneity in toxicity, organ damage, protein composition, and antivenom responsiveness. The differential abundance and isoform diversity of PLA_2_ and SVMP are key molecular bases for geographic variation in venom potency. These results emphasize the importance of considering venom geographic diversity in clinical treatment, pathogenesis research, and antivenom development. This study provides a theoretical and experimental foundation for improving the prevention and treatment of *D. acutus* envenomation and promotes the development of region-adapted antivenom strategies.

## Conclusion

In summary, *D. acutus* venoms from different geographic origins exhibit significant heterogeneity in toxicity, organ damage, and proteomic composition. This variation compromises the efficacy of commercial antivenoms, emphasizing the urgent need for region-adapted antivenom development to improve snakebite treatment.

## Supporting information

S1 Table Plasma biochemical parameters of mice injected with venom from 14 geographic regions of *D. acutus.* Mice were administered 0.2 × LD_50_ of regional venom via intraperitoneal injection. Blood samples were collected at 24 h post‑injection, and plasma biochemical parameters were measured, including ALT, AST, creatinine, CK, LDH, UA, CK‑MB, ALP, and FIB. Data are presented as mean values (three decimal places except for FIB).
(XLSX)

S2 Table Quantitative proteomic abundance of venom protein classes in D. acutus from 14 geographic regions. This table presents the relative quantitative abundance of identified venom proteins across 14 geographic populations of *D. acutus* in China (Anhui, Chongqing, Fujian, Guangdong, Guangxi, Guizhou, Hainan, Henan, Hubei, Hunan, Jiangxi, Shanghai, Yunnan, and Zhejiang). Proteins are grouped by functional categories, including C‑type lectins (Snaclec), cytoskeletal proteins, cytotoxins, enzyme inhibitors, growth factors, heat shock proteins, metabolic enzymes, metalloproteinases, neurotoxins, phospholipases A_2_, ribosomal proteins, serine proteases, and transport proteins.
(XLSX)

S1 Raw Images Original, uncropped gel images for SDS‑PAGE analysis (corresponding to Fig 4A). The file contains the original, uncropped SDS‑PAGE gel images for all 14 regional venom samples used in this study.
(PDF)
