# Supplementary material for: Geographic heterogeneity of Deinagkistrodon acutus venom: A multi-regional study of toxicity, proteomics, and antivenom efficacy
Source: PLoS Negl Trop Dis. 2026 Aug 11;20(8):e0014640. doi: 10.1371/journal.pntd.0014640 (PMC13480627; doi:10.1371/journal.pntd.0014640)
Supplement: Supplementary file 1 [file pntd.0014640.s003.pdf]

The methods have been described in detail in the manuscript.

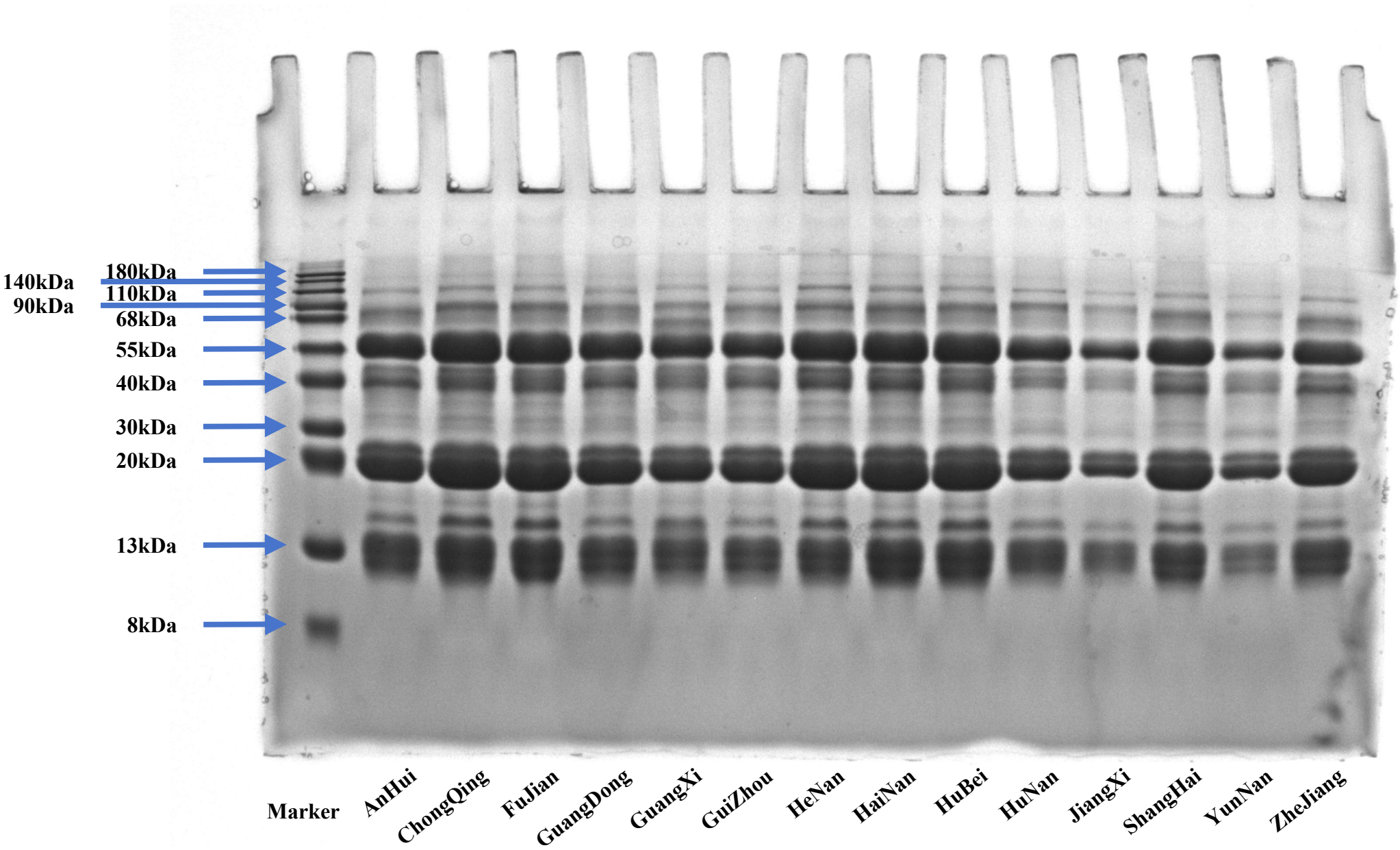

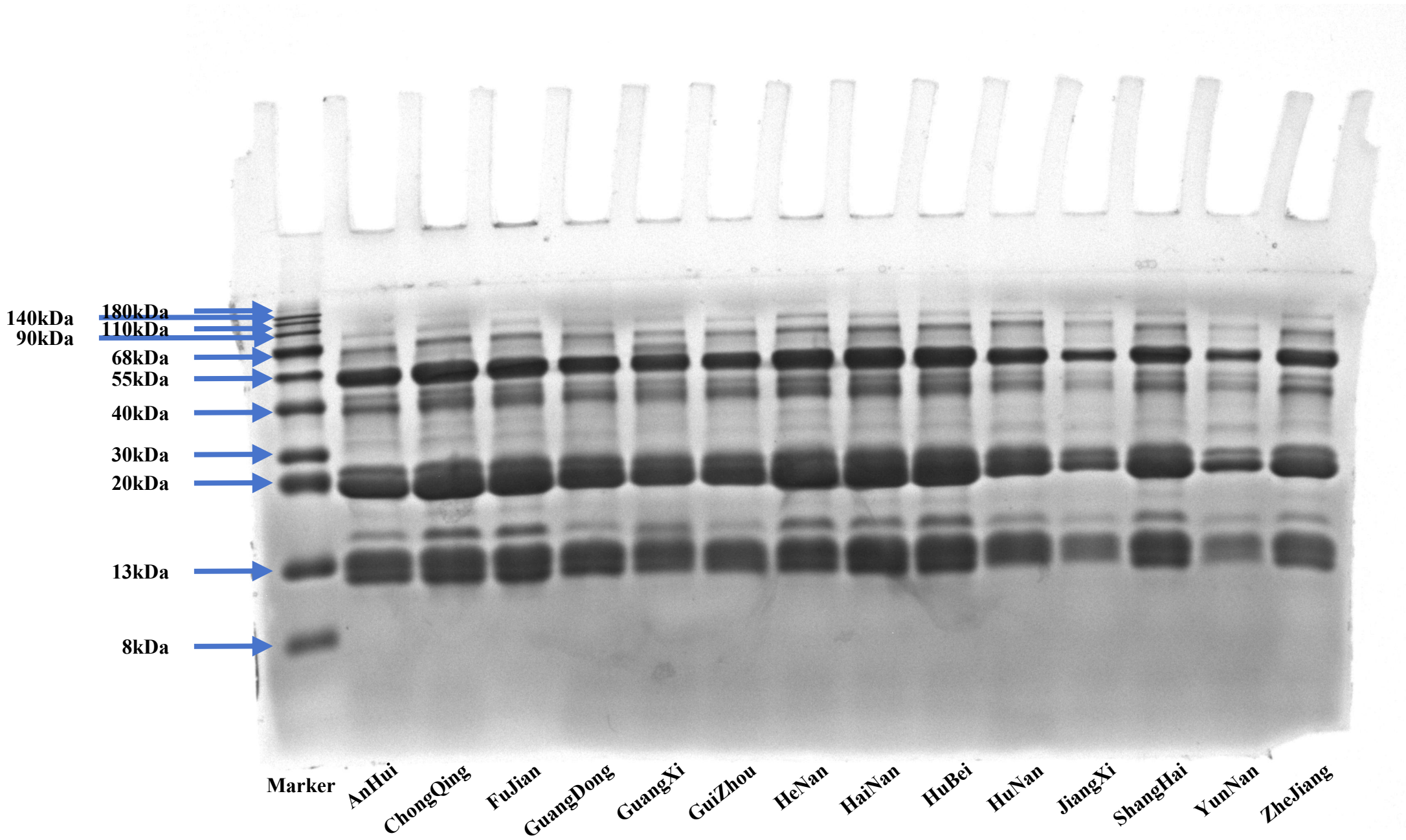
140kDa
180kDa
110kDa
90kDa
68kDa
55kDa
40kDa
30kDa
20kDa
13kDa
8kDa
Marker
AnHui
ChongQing
FuJian
GuangDong
GuangXi
GuiZhou
HeNan
HaiNan
HuBei
HuNan
JiangXi
ShangHai
YunNan
ZheJiang

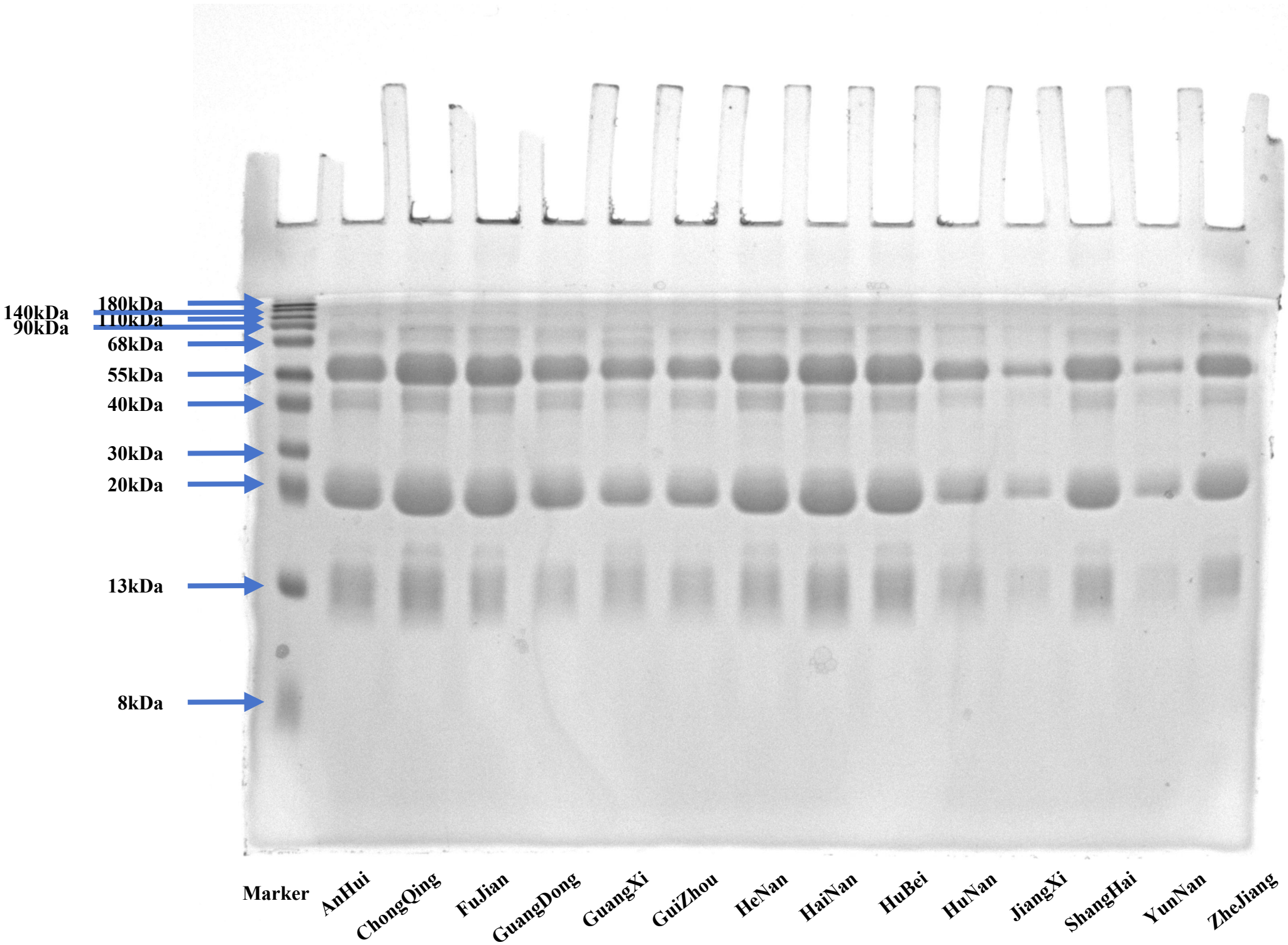

140kDa
90kDa
180kDa
110kDa
68kDa
55kDa
40kDa
30kDa
20kDa
13kDa
8kDa
Marker
AnHui
ChongQing
FuJian
GuangDong
GuangXi
GuiZhou
HeNan
HaiNan
HuBei
HuNan
JiangXi
ShangHai
YunNan
ZheJiang
